# The Role of Chemokines in Psoriasis—An Overview

**DOI:** 10.3390/medicina57080754

**Published:** 2021-07-26

**Authors:** Natalia Zdanowska, Marta Kasprowicz-Furmańczyk, Waldemar Placek, Agnieszka Owczarczyk-Saczonek

**Affiliations:** Department of Dermatology, Sexually Transmitted Diseases and Clinical Immunology, School of Medicine, Collegium Medicum, University of Warmia and Mazury, 10-229 Olsztyn, Poland; martak03@wp.pl (M.K.-F.); w.placek@wp.pl (W.P.); aganek@wp.pl (A.O.-S.)

**Keywords:** chemokines, psoriasis, systemic treatment

## Abstract

By participating in both the recruitment and activation of T lymphocytes, macrophages and neutrophils at the site of psoriatic inflammation, chemokines play an important role in the pathogenesis of psoriasis and, crucially, may be one indicator of the response to the systemic treatment of the disease. As a result of their major involvement in both physiological and pathological processes, both chemokines and their receptors have been identified as possible therapeutic targets. Due to their presence in the inflammatory process, they play a role in the pathogenesis of diseases that often coexist with psoriasis, such as atherosclerosis and psoriatic arthritis. Chemokines, cytokines and adhesion molecules may be biological markers of disease severity in psoriasis. However, the mechanism of inflammation in psoriasis is too complex to select only one marker to monitor the disease process and improvement after treatment. The aim of this review was to summarize previous reports on the role of chemokines in the pathogenesis of psoriasis, its treatment and comorbidities.

## 1. Introduction

Chemokines are a group of low-molecular-weight polypeptides that contain 60–100 amino acids [1]. They induce the chemotaxis of lymphocytes, monocytes and neutrophils according to their concentration gradients [1,2]. Chemokines do not have a single profile of influence; among them, there are those with inflammatory (CXCL1, 2, 3, 5, 6, 7 and 8), homeostatic (CXCL12 and 13) and both homeostatic and proinflammatory properties (CXCL9, 10, 11 and 16). CXCL4 is a plasmatic chemokine but can also be associated with platelets [3]. Chemokines play such an important role in the recruitment and activation of T cells, macrophages, and neutrophils in psoriatic inflammation that molecules that block their activity (e.g., CCL27, CCL20) are currently being investigated as potential therapeutic targets in psoriasis [2].

**Figure 1:**
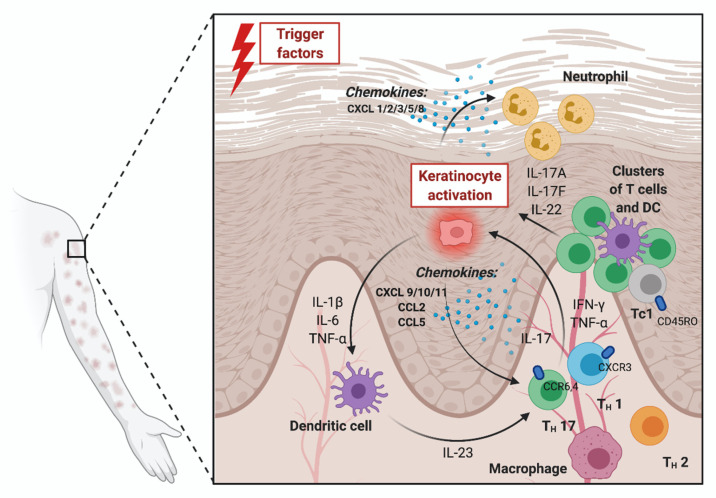
Presence of chemokines in the pathogenesis of psoriasis. Abbreviations: CCL—chemokine C-C motif ligand; CCR—chemokine C-C motif receptor; CXCL—C-X-C motif chemokine ligand; CXCR—C-X-C motif chemokine receptor; DC—dendritic cell; Th1—T helper 1 lymphocyte; Th17—T helper 17 lymphocyte; Th2—T helper 2 lymphocyte; IL—interleukin; IFN-γ—interferon gamma; TNF-α—tumor necrosis factor alpha. *Own design based on [3,4]; figure created with BioRender.com*.

The differentiation of naive T cells into effector cells, such as Th1 or Th17 lymphocytes, takes place in the regional lymph node and is induced by dendritic cells. The chemokine gradient promotes the migration of immune cells expressing CXCR3 or CCR3 receptors into the skin. Dendritic cells in the skin release interleukin (IL)-23, while other proinflammatory mediators are produced by Th 17 (IL-17A, IL-17F and IL-22) and Th1 (IFN-γ and TNF-α) lymphocytes [3]. Chemokines derived from keratinocyte effector cells activated by the aforementioned inflammatory mediators play a major role in maintaining leukocyte recruitment to inflammatory sites [4]. Clusters of dendritic cells and T lymphocytes around blood vessels are formed in the presence of chemokines produced by macrophages (CCL19), while CXCL1 together with IL-8 (CXCL8) is involved in attracting neutrophils to the epidermis [3]. The presence of chemokines in the pathogenesis of psoriasis (especially disease maintenance) is demonstrated in Figure 1 [4,5].

Many chemokines, in addition to psoriasis, are found in the pathogenesis of other inflammatory diseases and also represent potential therapeutic targets for them. This has been confirmed for generalized pustular psoriasis [6], psoriatic [7,8] and rheumatoid arthritis [9], cardiovascular disease [10,11], atherosclerosis [12], obesity [13], metabolic syndrome [14] and atopic dermatitis [15,16,17], irritant dermatitis [16], thyroiditis [18] or Schnitzler syndrome [19].

Table 1 summarizes the function and the role of selected chemokines in psoriasis, as well as the occurrence of chemokines among patients with comorbidities and psoriasis treatment impact on their serum levels.

### Systemic Treatment of Psoriasis and Its Impact on Chemokines

By participating in both the recruitment and activation of T lymphocytes, macrophages and neutrophils at the site of psoriatic inflammation, chemokines play an important role in the pathogenesis of the disease and, crucially, may also be one indicator of response to the systemic treatment of the disease.

Dai et al. evaluated the expression of MIP-1a, MIP-1b and MCP-1 in patients with psoriasis vulgaris compared with disease severity (measured via PASI) before and after 4-week treatment with oral acitretin along with topical tacrolimus. Peripheral blood levels of MIP-1, MIP-1 and MCP-1 were higher in psoriasis and positively correlated with PASI, whereas they were significantly decreased after treatment [1].

Abdelaal et al. described CXCL12/SDF-1 expression in skin biopsies of patients with psoriasis vulgaris and psoriatic arthritis (PsA) during 6-week methotrexate (MTX) therapy compared to healthy volunteers. PsA patients had significantly higher CXCL12 expression than patients with psoriasis vulgaris before treatment. There was a significant decrease in CXCL12 expression in patients with psoriasis vulgaris after MTX treatment. By contrast, there was no significant difference before and after treatment in patients with PsA [8]. Similarly, Quan et al. showed higher CXCL12/SDF-1 expression in psoriatic lesions compared to skin of healthy volunteers [47].

Abji et al. put forward the concept that CXCL10 may be involved in the pathogenesis of PsA and may be a potential predictive biomarker for the development of PsA in patients with psoriasis. Serum CXCL10 levels and gene expression in synovial fluid were significantly higher in patients who developed PsA [48]. Stoof et al. showed that in vitro fumaric acid esters dependently inhibited chemokine production (CXCL1, CXCL8, CXCL9, CXCL10) and CXCL11 transcription [33]. Another study found a rapid decrease in CXCL10 and CCL20 after 6-month treatment with etanercept [36]. In contrast, apremilast inhibited in vitro secretion of CXCL9, CXCL10, IFN-g, TNF-a and IL-2, IL-12 and IL-23 from human primary peripheral blood mononuclear cells [18,37].

Adhesion molecules play an important role in the migration of T lymphocytes to lesion sites in psoriasis [49]. In the pathogenesis of psoriasis (especially in its erythrodermic form), the increase in blood vessels and overexpression of ICAM-1 play an important role [50]. Examination of skin biopsy specimens showed increased expression of ICAM-1, vascular cell adhesion molecule 1 (VCAM-1) and endothelial selectin (E-selectin). Its intensity correlated positively with disease severity. At the same time, plasma plasminogen activator inhibitor type 1 (PAI-1) levels were elevated in psoriasis and correlated with increased angiogenesis [44]. PAI-1 is a single-chain glycoprotein belonging to the serine protease inhibitor family. Elevated PAI-1 levels are associated with abdominal obesity, insulin resistance, hypertriglyceridemia, thrombosis and cardiovascular disease, also recognized as frequent comorbidities of psoriasis. Increased plasma PAI-1 levels were observed in patients with psoriasis and decreased during therapy. Moreover, a positive correlation between elevated homocysteine and PAI-1 levels was found in psoriasis patients. It is worth noting that homocysteine may alter the binding of PAI-1 to the endothelium [51].

TREM-1 (CD354), belonging to the immunoglobulin superfamily, shows constitutive expression on peripheral blood monocytes and neutrophils. It is activated synergistically with TLR (toll-like receptor) agonists and interacts with an epidermal antimicrobial peptide (cathelicidin/LL37) and may be involved in both infection-induced and noninfectious inflammatory processes. Increased expression of TREM-1 in psoriasis was shown to decrease with effective treatment [46].

## 2. Conclusions

Chemokines are important in the pathogenesis of inflammation, but not all of them are proinflammatory. Their role in psoriatic inflammation is seen mainly in the maintenance phase. By acting on immune cells that have receptors for them, chemokines promote cell migration and inflammatory infiltration within psoriatic skin lesions. The studies presented above suggest that chemokines, cytokines and adhesion molecules may be biological markers of disease severity in psoriasis and its response to systemic treatment. However, the mechanism of inflammation in psoriasis is too complex to select only one marker to monitor the disease process and improvement after treatment.

## Figures and Tables

**Table 1:** Role and function of selected chemokines in psoriasis.

Molecule	Receptor	Function	Role in Psoriasis	Presence in Psoriasis Comorbidities	Impact of Psoriasis Treatment
CCL1/l-309	CCR8	- secreted by T cells, monocytes and mast cells; - attracts immature dendritic cells (monocytes) and activated Th2 type cells; - does not attract neutrophils or granulocytes [20]	- binds to CCR8 receptors on T cells and dendritic cells; - expressed mainly on dendritic cells, mast cells and endothelial cells of the dermis [20]	- Psoriatic arthritis [7] - Atopic dermatitis [15]	- n/a
CCL2/MCP-1	CCR2 CCR4	- attracts and activates monocytes; - attracts basophils, activated T cells, NK cells, and immature dendritic cells [20]	- produced by the keratinocytes; - synergistically amplified by TNF-α and IFN-γ [21]; - chemotactic signal given by monocytes via CCR2 leads to their differentiation into macrophages, which act as antigen-presenting cells and secrete TNF-α [21]	- Rheumatoid arthritis [9] - Schnitzler syndrome [19]	- Acitretin: ↓ [1] - NB-UVB phototherapy: ↓ [22,23] - Anti-TNF: ↓ [24] - Anti-CD11: ↑ [24]
MIP-1α/MIP-1β (CCL3/CCL4)	CCR1 CCR5	- chemoattractant for eosinophils, monocytes, B cells and immature dendritic cells; - activates macrophages; - CCL3 selectively attracts CD8 T lymphocytes and CCL4 CD4 T lymphocytes [20]	- levels of MIP-1α, MIP-1β are significantly increased in peripheral blood of psoriatic patients; - Th1 lymphocyte, dendritic cells and monocyte chemotaxis to the tissue of skin lesion by CCL4 [1,25]	- Generalized pustular psoriasis [6] - Metabolic syndrome [14]	- Acitretin: ↓ [1]
CCL5/RANTES	CCR1 CCR3 CCR5	- chemoattractant for eosinophils, basophils, monocytes, effector memory T cells (CD4^+^/CD45RO^+^), B cells, NK cells and immature dendritic cells [20]	- highly expressed by keratinocytes in psoriatic lesions (induction of the expression by proinflammatory cytokines such as IFN-γ and TNF-α); - recruitment of leukocytes to the skin [25,26]	- Atopic dermatitis [16] - Irritant contact dermatitis [16]	- UVB phototherapy: ↓ [23] - NB-UVB phototherapy: ↓ [27]
CD40L/TNFSF5	CD40	- regulation of cell proliferation, apoptosis and proinflammatory properties, primarily through the induction of NF-κB [28]	- binding of CD40L to CD40 enhances proliferation, differentiation and activation of B lymphocytes; - important factor maintaining the autoimmune process [29]	- Obesity [13] - Type 2 diabetes mellitus [13] - Atopic dermatitis [17]	- Apremilast: ↓ [30]
CXCL1/GRO-α	CXCR2	- significant role in regulating wound healing and inflammation by recruiting neutrophils to inflammatory sites [31]	- neutrophil chemotaxis to the skin [25]	- Endothelial impairment [10]	- Bath-PUVA phototherapy: ↓ [32]. - Dmethylfumarate: ↓ [33].
CXCL10/IP-10	CXCR3	- secreted by lymphocytes, monocytes, keratinocytes, fibroblasts and endothelial cells in response to IFNγ and TNFα; - recruits T cells, eosinophils, monocytes and natural killer cells to sites of inflammation; - angiostatic properties [34,35]	- present in keratinocytes and the dermal infiltrate of active psoriatic plaques; - decreased expression after successful treatment [18]	- Psoriatic arthritis [7] - Autoimmune thyroiditis [18]	- Dmethylfumarate: ↓ [33]. - Anti-TNF: ↓ [24,36]. - Apremilast: ↓ [37]
CXCL11/I-TAC	CXCR3	- production induced by IFN-γ; - up-regulated in IFN-γ-treated monocytes, bronchial epithelial cells, neutrophils, keratinocytes and endothelial cells; - recruitment of T lymphocyte recruitment to sites of inflammation [38]	- expressed in psoriatic lesions along with macrophages and T-cells; - mainly expressed by basal keratinocytes [39]	- Alopecia areata [40] - Atopic dermatitis [17]	- Dmethylfumarate: ↓ [33].
CXCL12/SDF-1	CXCR4 CXCR7	- attraction of T cells, monocytes, neutrophils and promotion of T cells adhesion to ICAM-1 [41]	- neutrophil, monocyte, lymphocyte and dendritic cells chemotaxis [8]; - keratinocyte proliferation via ERK activation [42]	- PsA: significantly higher expression before treatment but not after treatment [8]	- MTX: ↓ [8]
Serpin E1/PAI-1	tPA uPA	- increases angiogenesis; - potent inhibitor of fibrinolysis; it interacts with tissue-type and urokinase-type plasminogen activator, leading to inhibition of the conversion of plasminogen to plasmin [43]	- increases angiogenesis in psoriatic lesions [44]	- Cardiovascular risk [12] - Metabolic syndrome [44]	- NB-UVB photoherapy: ↑ [45] - Anti-TNF: ↓ [45].
TREM-1	n/a	- member of the immunoglobulin superfamily; - expressed on monocytes and neutrophils in peripheral blood, airway epithelial cells, hepatic endothelial cells, NK cells, dendritic cells, B and T cells; - activated synergistically with TLR agonists and interacts with epidermal antimicrobial peptide (cathelicidin/LL37); - activation results in the production of proinflammatory cytokines, such as MCP/CCL2, MIP-1α, TNF and IL-8, inducing innate and adaptive immune responses [46]	- activation of the TREM-1 receptor (by its unknown ligand) leads to DAP12 recruitment and production of cytokines (MCP/CCL2, IL-8 and TNF); - expressed on circulating neutrophils (producing IL-17) in both normal and psoriatic subjects; - role in early inflammatory processes [46]	- Cadriovascular disease [11]	- NB-UVB phototherapy: ↓ (responders) [46] - Anti-TNF and anti-IL-17: ↓ [46].

Abbreviations: CCL—chemokine C-C motif ligand; CCR—chemokine C-C motif receptor; CD40L—CD40 ligand; CXCL—C-X-C motif chemokine ligand; *CXCR*—C-X-C motif chemokine receptor; GRO-α—growth-regulated oncogene *alpha*; *DAP12*—DNAX-activating protein of 12 kDa; *I-TAC*—interferon-inducible T-cell alpha chemoattractant; *ICAM-1*—intercellular adhesion molecule-1; IFN-γ—interferon gamma; IP-10—interferon gamma-induced protein 10; MCP1—monocyte chemoattractant protein 1; MIP—macrophage inflammatory protein; MTX—methotrexate; NK—natural killer cells; *NF-κB*—nuclear factor kappa-light-chain-enhancer of activated B cells; NB-UVB—narrow band ultraviolet B; PAI-1—*plasminogen activator inhibitor type 1*; PsA—psoriatic arthritis; PUVA—Psoralen Ultra-violet A; RANTES—regulated on activation, normal T-cell expressed and secreted; SDF-1—*stromal cell-derived factor 1*; TLR—toll-like receptors; TNF-α—tumor necrosis factor alpha; *tPA*—tissue plasminogen activator; *TREM-1*—triggering *receptor* expressed on myeloid cells 1; uPA—urokinase plasminogen activator; ↓—decrease; ↑—increase.

## Data Availability

Not applicable.
